# A Path Analysis of the Effect of Neighborhood Built Environment on Public Health of Older Adults: A Hong Kong Study

**DOI:** 10.3389/fpubh.2022.861836

**Published:** 2022-03-14

**Authors:** Shuangzhou Chen, Ting Wang, Zhikang Bao, Vivian Lou

**Affiliations:** ^1^Department of Social Work and Social Administration, Faculty of Social Sciences, The University of Hong Kong, Hong Kong, China; ^2^Sau Po Center on Ageing, The University of Hong Kong, Hong Kong, China; ^3^Division of Landscape Architecture, Department of Architecture, The University of Hong Kong, Hong Kong, China; ^4^Department of Real Estate and Construction, Faculty of Architecture, The University of Hong Kong, Hong Kong, China

**Keywords:** older adults, public health, neighborhood built environment, path analysis, Hong Kong

## Abstract

**Introduction:**

Health deterioration among frail older adults is a public health concern. Among the multi-dimensional factors, the neighborhood built environment is crucial for one's health. Although the relationship between the built environment and health in the general population has been thoroughly investigated, it has been ignored in the case of frail older adults, who may have difficulties in their daily basic living skills. A path analysis is constructed to model the proposed theoretical framework involving the neighborhood built environment and health among frail older adults. This study thus aims to investigate the environmental influences on health, and to validate the theoretical framework proposed for health and social services.

**Methods:**

This study used secondary data collected in Hong Kong. A sample of 969 older community dwellers aged 60 or above were frail with at least one activity of daily living. Demographic information, neighborhood built environment data, service utilization, and health conditions were collected from these participants and their caregivers. A path analysis was performed to examine the proposed theoretical framework.

**Results:**

The health condition was of general concern, including frailty and incapacities in daily activities in frail older adults. Besides psychosocial factors, service use, and caregivers' care quality, the built environment had a significant impact on the health of older adults as well. Specifically, more facilities offering services and groceries, a shorter distance to the nearest metro station, and more greenery exposure are associated with a better-expected health condition among frail older adults.

**Discussion:**

The proposed theoretical framework successfully supplements past negligence on the relationship between the built environment and the health of frail older adults. The findings further imply that policymakers should promote the usability of transit and greenery in neighborhoods and communities. In addition, service utilization should be improved to meet the basic needs of frail older adults in the communities.

## Introduction

In Hong Kong, adults aged 60 and over represented more than one-sixth of the total population, while the prevalence of aging is estimated to exceed 30% in 2041 (1). Among this aging population, the prevalence of frailty with underlying health conditions is reportedly more than 50% (2, 3). Frail older adults are identified as being weak, with complex medical problems, impaired mental abilities, compromised ability for independent living, and often in need of assistance for daily activities (4). Worsening health and preventive risk factors have traditionally been priorities in public health policy for frail older adults (5, 6). Declining health is a complex state involving multi-dimensional aspects (7). Commonly used indices measuring health conditions include frailty level, activities of daily living, and instrumental activities of daily living. The frailty level refers to physical, psychological and social aspects (8). Activities of daily living refer to a person's basic functional status in daily living (e.g., eating); while instrumental activities of daily living requires a higher extent of functional skills (e.g., financial management) in daily living scenarios (9).

Among all the preventive factors for deteriorating health, several critical components are the necessity to facilitate improvement in the health of frail older adults. The health promotion system will not function well without these critical components: the neighborhood built environment, and two intermediate factors—service utilization and caregivers' quality of life. Studies have shown that the neighborhood built environment among potential risk factors plays a decisive role cause declining health in frail older adults (10). The significance of the neighborhood built environment in health studies has drawn the noticeable attention of public health and gerontology scholars over the past few decades (11, 12). For example, a deficiency of environmental greenery has been reported to adversely affect residents' health and daily living capacities (13). The paucity of social services facilities is considered to compromise one's living conditions, while deficits in mobility and transportive approaches limit people's walkability and physical mobility (14, 15). On the contrary, sufficiently available greenery, social service and grocery facilities, and transit in a sustainable society with well-designed urban planning are greatly beneficial to the physical, psychological, and social aspects of human beings in their daily living scenarios (10). Moreover, professional services, such as Integrated Home Care Services, have been initiated and planned by the Hong Kong government and Social Welfare Department for frail older adults (16). Despite the expected values of services provided in the neighborhood built environment, the services do not fully meet the needs of frail older adults and are poorly utilized by the targeted frail aging population if their caregivers are not involved (17). Frail older adults commonly have impaired independent living abilities and are dependent on assistance to perform their daily activities (18). Caregivers are the main workforce among formal and informal caregivers to take care of frail older adults and support their frailty in their physical mobility, psychological stability, and social interactions (19). Therefore, with the aids from caregivers in the family, frail older adults are able to access the resources in the neighborhood environment and take advantages of services that are available to them in the communities. In this sense, caregivers' quality of life determines their care quality and eventually benefit the frail older adults. Poor quality of life is generally associated with low care quality provided and declining health among frail older adults (18).

To sum, the existing literature fails to tackle two prominent concerns in this proposed framework integrating neighborhood built environment factors, intermediate factors, and health outcomes of frail older adults. First, there has been less recognition of the importance of the built environment in the neighborhood in relation to the intermediate variables and the health outcomes of frail older adults. Second, factors contributing to the health conditions of frail older adults have been investigated sporadically without comprehensive theoretical guidance. Therefore, this study aims to 1) investigate whether the neighborhood built environment influences the service use and health; 2) validate a theoretical framework proposed to interpret frail older adults' health conditions that take into account environmental factors, social services use, and caregivers' quality of life by using the path analysis method.

## Theoretical Background

The relationship between the neighborhood built environment and frail older adults' health has been weakly established due to critical negligence of intermediate components linking the neighborhood environment and health. To best develop a practical conceptual framework, that balances between robustness and parsimony in theoretical development, the Andersen's Model and Stress-Adaptation Model are integrated into our proposed theoretical framework with two essential intermediate components: service use and caregiver's quality of life.

The Andersen's Model is commonly known as the Andersen healthcare utilization model. This model has been an essential framework for diagnosing factors associated with the use of healthcare services (20). This overarching health model underscores the importance of service use to sustain community health and offers potential policy and clinical implications in promoting frail older adults' health conditions. The health condition of frail older adults, according to the Andersen's Model, comprises three aspects: frailty, activities of daily living (ADL), and instrumental activities of daily living (IADL). Frailty in older adults is often accompanied with a lower level of physical and social activities, worse body control, slowed cognitive reactions, physical and cognitive fatigue, and unforeseen weight loss (21). Besides frailty, these older adults also suffer from challenges in their daily life. ADL and IADL are the major indices to measure such challenges. ADL mainly refers to self-care tasks, which are the necessary things that people have to do daily, from getting up in the daytime till going to bed in the nighttime. Given various versions of ADL measurements, the ADL items are commonly identified as in the following: get up from bed or out of a chair, get dressed, personal and toilet hygiene, bathing and showering, eating, walking, climbing stairs, and other responses to safety (22, 23). On the other hand, IADL was identified for older community dwellers and its items relate to one's independent living abilities that enable one to walk out of home or take care of oneself independently, such as cooking for oneself, taking medicines on time, doing grocery or laundry, paying bills and going to banks on one's own (23, 24).

The Andersen's Model categorizes influential factors into three parts: predisposing factors, enabling factors, and need factors. Predisposing factors usually refer to sociodemographic characteristics that direct one's perception of service utilization. Enabling factors refer to resources or capacities that influence service utilization. Need factors refer to one's needs and demands for services due to illness or impairment (e.g., positive or negative attitude) (25, 26). In the context of older adults with frailty and inabilities in daily activities, our “predisposing factors” should at least include demographic information such as age, gender, educational level, and living arrangement. It is indicated that older adults from diverse demographic groups have different behaviors in service use. For example, female older adults are more aware of using available services to benefit their health, whereas males receive services when they desperately need them (27). Old olds, in general, use the services more often than young olds (28). Studies also show that people with a higher level of educational attainment are more likely to use services (29). The “enabling factor” refers to caregivers' perceptions of stress in their daily caregiving. Since caregivers frequently perceive caregiving stress, counseling and respite services may help alleviate their anxiety and psychological burden (30). The “need factor” refers to the needs for services provided to both frail older adults and their caregivers. The need for services is crucial in the process of service utilization. Community and social services sometimes may not appropriately address the actual expressed needs from both frail older adults and caregivers, leading to unmet needs and worsened life experiences among the population as well as the low cost-effectiveness of community services (25). More frequent service use is found among older adults and their caregivers with high service needs (31). Frail older adults have basic needs for assistance with their daily living activities, such as dining, bathing, and mobility, as well as some activities that require an advanced cognitive functioning level, such as financial management and commuting (9). Even though the neighborhood built environment was not explicitly included in the earlier version of the Andersen's Model, recent empirical studies emphasized the necessity to associate service utilization with environmental factors in building a sustainable society (32). Neighborhoods with functioning social organizations and desirable accessibility show high utilization of services for frail older adults with complex needs dwelling at home (33). In addition, the built environment, such as transportation and green spaces, affect local health care utilization and residents' health and wellbeing (34).

In sustaining a decent level of health, according to the Stress-Adaptation Model, the caregiver's quality of life (QoL) is another crucial intermediate component as the caregiver is an irreplaceable agent in our proposed health framework. For daily activities, frail older adults who have lost their independent living abilities heavily depend on caregivers in their daily activities (18). Caregivers, being the backbone among all formal and informal caregivers, have extra responsibilities beyond their own personal life, such as providing emotional support, accompanies in daily dining, toileting, commute, and activities, contacting medical professionals, tracking treatment and medications, coordinating care with other family members, keeping family and relatives informed, making financial and legal arrangement (35). Consequently, these caregivers suffer greatly from physical exhaustion, psychological stress, and social isolation as their lives are occupied by daily care responsibilities (36). Moreover, caregiving stress and burden lead to dissatisfaction with the support or services provided (37), or unwillingness to seek the services because they prioritize the services for frail older adults over the ones for themselves (38). Besides, stressed caregivers broadly report a low QoL (39). More importantly, some caregivers with deteriorated QoL develop severe mental disturbance, such as depression or anxiety, which further cause mental breakdown and inabilities to care for their frail older family members any longer (40, 41). Therefore, The intertwined QoL of caregivers and the health of frail older adults can easily develop a vicious cycle, in which worse QoL leads to declined health owing to worse care quality, whereas a miserable health condition increases more caregiving stress that adversely affects QoL (18). The Stress-Adaptation Model also assumes caregivers, who inevitably experience high levels of stress with a lower QoL, may have compromised quality of care and expect an adverse health outcome among their frail care recipients (42). Empirical studies demonstrate that improved QoL of caregivers results in better care and reduced incidences of frailty and functioning incapacities in the daily activities of their frail care recipients (18, 43, 44).

In summary, by adopting the Andersen's Model and the Stress-Adaptation Model, our proposed theoretical framework incorporates the neighborhood built environment while involving intimate caregiver partners to interpret the potential factors influencing the health condition of frail older adults. The intermediate variables, service uses and caregivers' QoL, associated with health outcomes can be jointly attributed to individual and environmental factors. Neighborhood built environmental factors also contribute to improved use of services and better QoL of caregivers. Improved use of services and QoL of caregivers are found associated with greenery, social service and grocery facilities, whereas the distances to the transit discourage service use and lower caregiver's QoL (13, 17). Supported by the aforementioned Andersen's Model for health services and the Stress-Adaptation Model on caregivers' QoL, this study aims to gain a clear understanding on how the neighborhood built environment influences the service use and health.

## Methods

### Study Setting and Participants

This study used secondary data derived from a large project undertaken in Hong Kong. The study with the original data investigates the health condition and service needs of Hong Kong older community dwellers and their family caregivers in 2018 (45). Participants were recruited from all districts in Hong Kong via (1) the community centers, and (2) companies and social organizations. Structured questionnaires administered by trained researchers were distributed to collect quantitative information. Demographics and health conditions of both the family caregiver and the CR in each dyad were interviewed and reported by the caregivers.

A total of 969 family caregivers were selected for this study by our preset criteria, which include 1) the CRs (care recipients—older adults) were aged 60 or above, 2) the CRs had one or more difficulties in both the Activities of Daily Living (ADL) and Instrumental Activities of Daily Living (IADL) lists since the past 3 months, and 3) they were the primary family caregivers of the CRs. On the other hand, the CRs aged below 60, had no difficulty in ADL and/or IADL, or had no caregiver were excluded from our study.

### Measures

**Figure 1:**
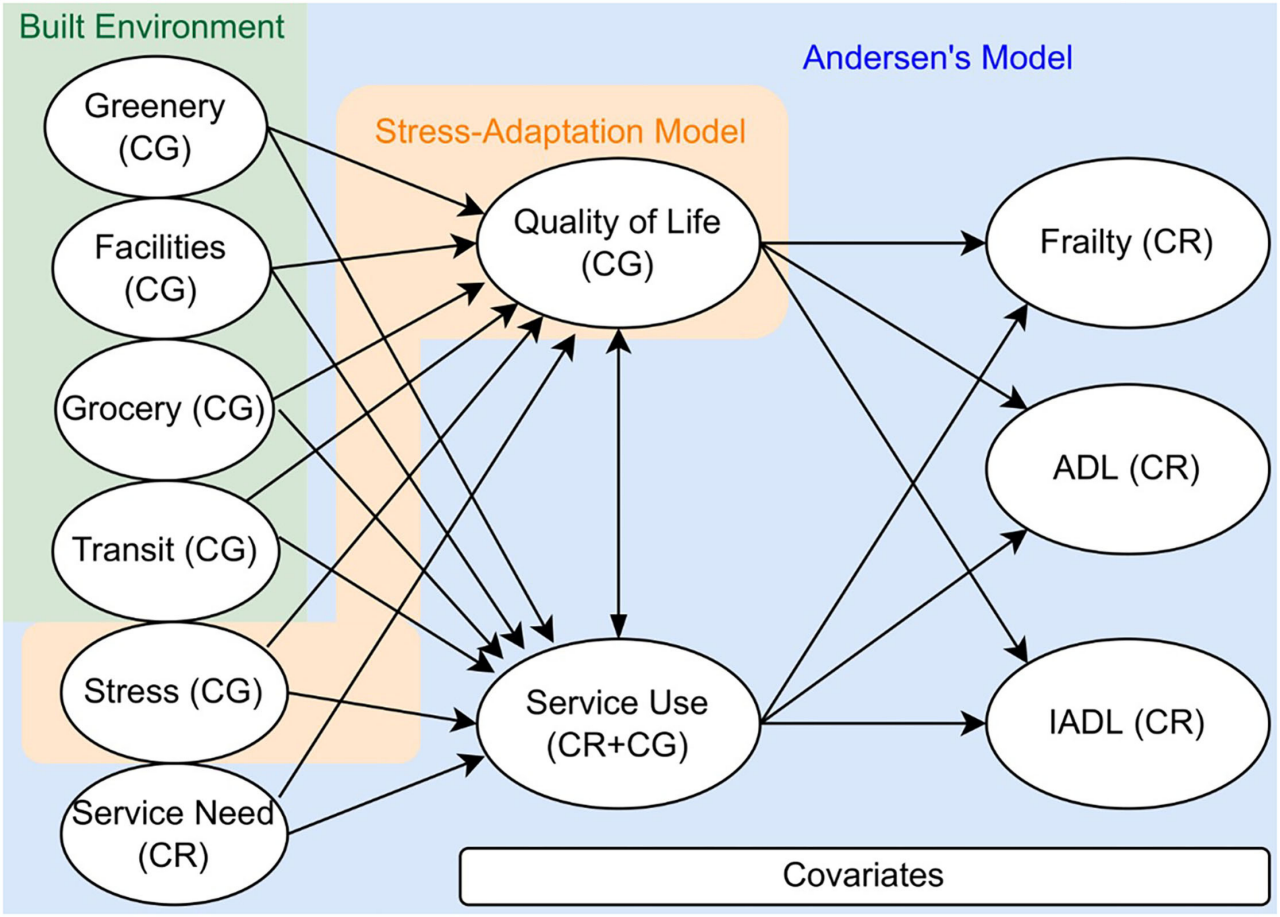
Conceptual model.

The measurements in this study cover outcome variables, intermediate variables, neighborhood variables, and individual variables following an integration of the Andersen's Model and the Stress-Adaptation Model (Figure 1).

#### Outcome Variables: Health Conditions

The health conditions in this study included two major components: activities of daily functioning and frailty level. To begin with activities of daily functioning, ADL being one of the essential scales measured one's abilities in engaging in activities of daily living, comprising personal hygiene, toileting, eating, etc. whereas, IADL measured one's abilities in performing more advanced or instrumental activities of daily living, including financial management, food preparation, laundry, etc. (46, 47). ADL included 6 items on a two-point scale (1—need assistance to do it; 0—can do it independently); and IADL included 11 items on a two-point scale (1—need assistance to do it; 0—can do it independently). Second, to measure one's frailty level, the frailty scale was used to consist of 16 items that separately evaluated physical, cognitive, psychological and social aspects of frailty, such as falling history, memory loss, depressiveness, and insufficient social support. The frailty scale used a three-point Likert scale including options of 0 (no such symptom), 1 (sometimes), and 2 (very much likely). Lower scores in the above-mentioned three scales indicated better conditions of ADL, IADL, and frailty.

#### Intermediate Variables: Quality of Life and Service Use

Two critical intermediate variables that lead to sustainable health conditions included quality of life (QoL) and service use. The QoL was measured based on the perceived adaptation level of caregivers to balance their life and care tasks. The QoL used a one-item question with 6 options ranging from 1 (well-balanced life) to 6 (hardly balanced life). A lower score indicated better QoL.

Service use condition was evaluated using a six-point scale with two options (1—have used the service; 0—have not used the service). Services covered community-provided paid and unpaid programs that facilitate emotional support, rehabilitation, counseling, nursing skills, financial assistance, and employment training for older adults and their caregivers. A higher score indicated more frequent use of services.

#### Neighborhood Built Environment Variables

The neighborhood built environment variables refer to the physical and built environment in the proximity of the residence. First, the physical and built environment consisted of the commonly interactive environment such as greenery in the living environment, social service facilities (e.g., retail, banks, post offices, rehabilitation and daycare centers, etc.), grocery (e.g., markets, shopping, restaurants, etc.), transit (e.g., buses, metro, taxis, etc.), and greenery (green spaces in public area, parks, and open grounds). While each participant's residential place was recorded as coordinates in the GIS system, the neighborhood built environment was analyzed in the vicinity of the given coordinates (48). For instance, the service facilities were measured by counting the number of facilities in the vicinity of 1,000 m. The grocery variable also recorded the total amount of markets, shopping, restaurants within the 1 km range away from each participant. For transit, the distance of the nearest metro station to one's residence was calculated. The greenery index was measured using google street view photos at a 100-meter interval covering each resident's neighborhood in a radius of 1 km. Within each extracted panoramic photo, the proportion of greenery area over the overall area in pixels was calculated using the machine learning technique (49–52).

#### Individual Variables: Caregiving Stress and Service Need

Caregiving stress measured the stress and burden perceived during one's caregiving experiences. Caregiving burden and stress were measured using the short version of the Zarit Burden Interview (ZBI-4) (53, 54). ZBI consisting of 4 items on the ratings of the 5-point scale (1—“never” to 5—“very often”) had scores ranging from 4 to 20. Higher scores indicated greater stress perceived.

Service need was measured using a need list of items whether the older adults wanted to address. The items in the need list included nine common illnesses such as arthritis, mild cognitive impairment, high blood pressure, etc. Two options were provided: 1—need for services, and 0—no need for service.

#### Covariate Variables

The covariate variables for caregivers included age (in years), gender (1—female, 2—male), and education attainment (1—none, 2—primary diploma, 3—secondary diploma, 4—college diploma, 5—graduate diploma or above). On the other hand, the older adults (CRs) had their age (in years), gender (1—female, 2—male), and homestay (0—living separately, 1—living together with the caregiver) were collected.

### Statistical Analyses

Descriptive statistics of the covariate, individual, environmental, and intermediate variables of both older adults and their caregivers were analyzed. To have a comprehensive view on the overall model, a path analysis was used to explore the relationships between the outcome, intermediate, environmental, individual, and covariate variables. Path analysis is a form of multiple regression statistical analysis method to modeling relationships between multiple dependent variables and multiple layers of independent variables. Partial regression coefficients were estimated for causal relationships between variables. Being a subtype of structural equation modeling, the exogenous and endogenous effects were both analyzed for detecting variances overly explained by covariate variables. The exogenous and endogenous effects were eventually eliminated according to the preliminary results showing that the variances explained by covariate variables majorly contributed to the path analysis model. We used R (version 4.1.0) to estimate this path analysis model with the adjusted maximum likelihood method.

## Results

### Sample Characteristics

According to the descriptive statistics in our findings, the older adults had an average age of 81-year-old (SD = 8.33), more males (62.4%) than females, tended to live together with their family members (95.0%). For their caregivers, the average age was 70.1 (SD = 10.8), there were more female caregivers (80.5%; including spouses and daughters). Educational attainment of caregivers were predominantly secondary school or below (90.2%) (Table 1).

**Table 1:** Health outcome and preventive factors.

**Key variables**	**Mean (SD)**	**Median [Min, Max]**	***N* (%)**
**Outcome variables**				
Frailty (CG)	14.5 (4.40)	14.0 [3.00, 27.0]	
ADL (CG)	4.51 (1.73)	5.00 [1.00, 6.00]	
IADL (CG)	7.11 (2.53)	8.00 [1.00, 10.0]	
**Intermediate variables**				
Quality of life (CG)	3.07 (2.02)	3.00 [1.00, 6.00]	
Service use (CR)	1.07 (1.13)	1.00 [0, 5.00]	
**Environment variables**				
Service facilities (GR)	10.1 (7.67)	9.00 [0, 33.0]	
Grocery (GR)	28.5 (26.2)	25.0 [0, 135]	
Transit distance (GR)	606 (959)	587 [1.06, 2086]	
Greenery (GR)	0.266 (0.169)	0.229 [0, 0.856]	
**Individual variables**				
Caregiving stress (CG)	12.5 (5.18)	12.0 [5.00, 25.0]	
Service need (GR)	3.72 (1.78)	4.00 [0, 9.00]	
**Covariate variables**				
*Frail older adults (CR)*				
Age	81.1 (8.33)	82.0 [58.0, 105]	
Gender				
Female			364 (37.6%)
Male			605 (62.4%)
Living arrangement				
Living separately			48 (5.0%)
Living together			921 (95.0%)
*Caregivers (CG)*				
Age	70.1 (10.8)	71.0 [29.0, 100]	
Gender				
Female			780 (80.5%)
Male			189 (19.5%)
Education				
None			109 (11.2%)
Primary			388 (40.0%)
Secondary			377 (38.9%)
College			85 (8.8%)
Graduate or above			10 (1.0%)

*CG, caregiver; CR, care recipient/frail older adults; GR, both frail older adults and caregivers*.

At the individual level, ZBI was scored 12.5 (SD = 5.18), indicating moderately high caregiving stress perceived by the caregivers. The service need from the older adults was counted around 4 (SD = 1.78), indicating a moderate need for services. At the environment level within 1 km range of the participants' residence, there were 10 (M = 10.1, SD = 7.67) facilities providing various social services, 28 (M = 28.5, SD = 26.2) places for groceries, 606 meters of distance away from the nearest metro station (SD = 959), 26.6% of greenery exposure in the neighborhood (SD = 16.9%). At the intermediate level, caregivers' QoL score was 3.07 (SD = 2.02), indicating a moderate level of life quality. Their service use condition was 1.07 (SD = 1.13), indicating a low level of service use.

The health conditions of the older adults were generally moderately worrying. Their frailty was scored 14.5 (SD = 4.40), indicating a moderate frailty level. They had moderately high scores in both ADL (M = 4.51, SD = 1.73) and IADL (M = 7.11, SD = 2.53).

### Correlational Analysis

**Figure 2:**
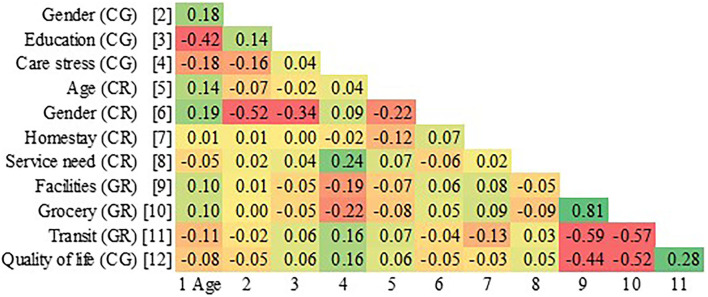
Correlational matrix. CG, caregiver; CR, care recipient/frail older adults; GR, both frail older adults and caregivers.

A correlational analysis was performed to examine collinearity between independent variables and verify the validity of using these variables in the same model (Figure 2). Collinearity is a critical issue to avoid as it can cause biased estimations in path analysis using any regression method. The correlational result showed that the absolute value of correlational coefficients among all variables ranged between 0 and 0.52, indicating mild to moderate collinearities. For the inherently highly correlated neighborhood built environment variables, their correlational coefficients were still acceptable ranging from 0.44 to 0.81. Therefore, these independent variables were all included conditionally in our path analysis.

### Path Analysis

The path analysis model was preliminarily evaluated with the goodness-of-fit estimations (Table 2). The path analysis used the unconstrained and box-constrained optimization in the maximum likelihood estimation. This path analysis indicated a robust model (*p* < 0.001). To measure the model fit, we used various indices, including Akaike's information criteria (24090.99), Bayesian information criteria (24236.44), Sample-size adjusted Bayesian information criteria (24144.33), root mean square error of approximation (0.078), standardized root mean square residual (0.046), comparative fit index (0.957), and Tucker-Lewis index (0.873). All these indices were at an acceptable level.

**Table 2:** Model statistics.

**Overall statistics**	
Estimator	ML
Optimization method	NLMINB
Number of model parameters	30
Number of observations	969
**Model index**	
Test statistic	1002.829
Degrees of freedom	45
*P*-value (Chi-square)	< 0.001
**Model fit index**	
Akaike (AIC)	24090.99
Bayesian (BIC)	24236.44
Sample-size adjusted Bayesian (aBIC)	24144.33
Root Mean Square Error of Approximation (RMSEA)	0.078
Standardized Root Mean Square Residual (SRMR)	0.046
Comparative Fit Index (CFI)	0.957
Tucker-Lewis Index (TLI)	0.873

**Figure 3:**
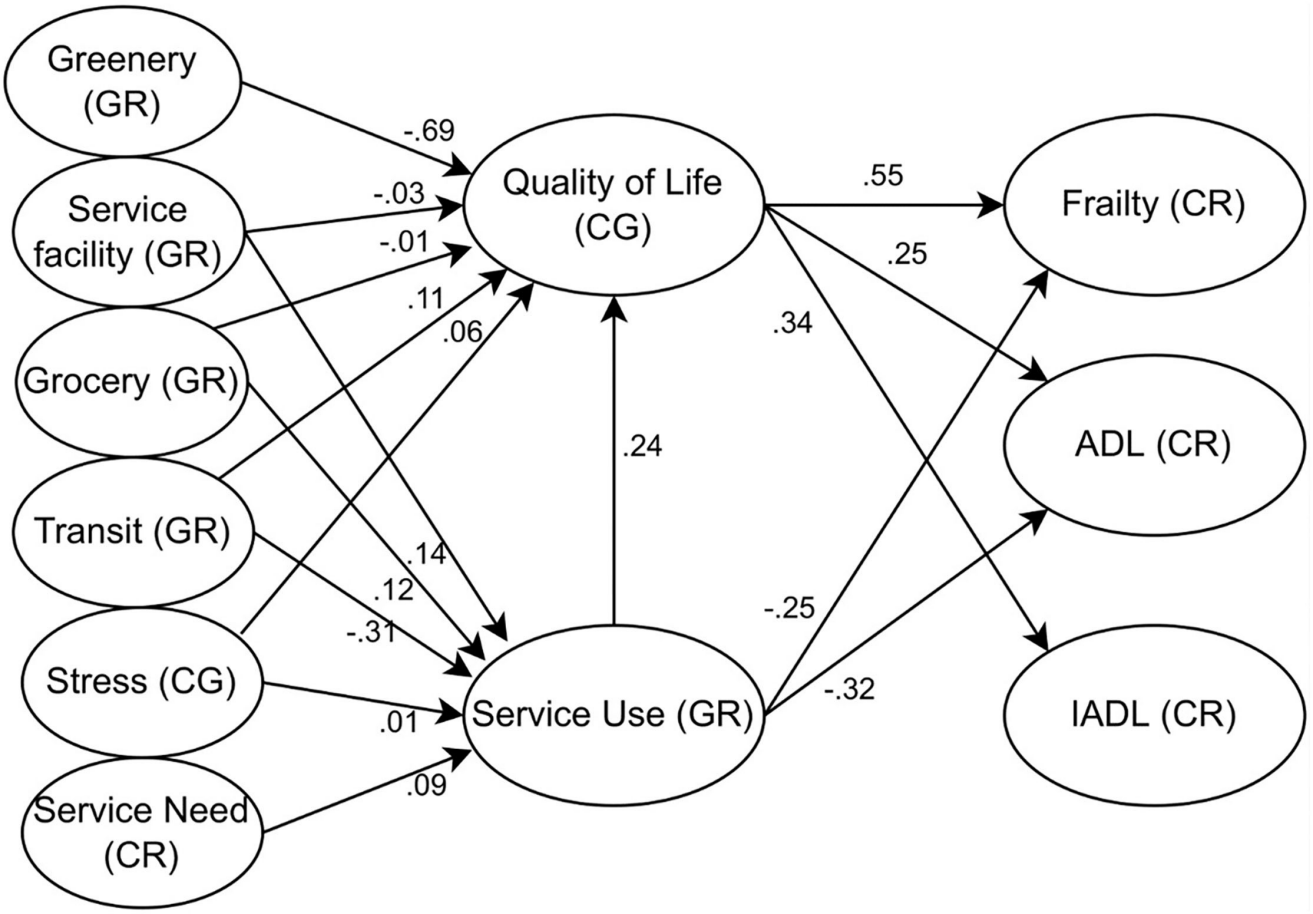
Statistical model. CG, caregiver; CR, care recipient/frail older adults; GR, both frail older adults and caregivers.

The results analyzed the direct effects of all paths within the path analysis model. Coefficients for all paths were shown in Tables 3, 4. The statistical framework, only showing significant paths, was demonstrated in Figure 3. There were two major layers in this path analysis: 1) environment, individual, and covariate variables predicting intermediate variables (Table 3); and 2) intermediate variables predicting outcome variables (Table 4).

**Table 3:** Path analysis—function of intermediate variables.

**IV**	**Intermediate variables**			
	**QoL (CG)**		**Service use**	
	**B**	***P***	**B**	***P***
**Intramediate relation (GR)**				
Service use	0.24	***		
**Environment variables (GR)**				
Facilities	−0.03	*	0.14	*
Grocery	−0.01	**	0.12	*
Transit	0.11	*	−0.31	*
Greenery	−0.69	*	0.19	
**Individual variables**				
Service need (CR)			0.09	***
Care stress (CG)	0.06	*	0.01	*
**Covariates (CG)**				
Age	−0.02	***		
Gender	−0.66	***		
Education	−0.07			
**Covariates (CR)**				
Age			0.06	*
Gender			−0.05	*
Homestay			0.03	*

CG, caregiver; CR, care recipient/frail older adults; GR, both frail older adults and caregivers. *P < 0.05, **P < 0.01, ***P < 0.001.

**Table 4:** Path analysis—function of health outcomes variables.

**IV**	**Outcome variables**					
	**Frailty**		**ADL**		**IADL**	
	**B**	***P***	**B**	***P***	**B**	***P***
**Intermediate variables**						
Quality of life (CG)	0.55	***	0.25	***	0.34	***
Service use (GR)	−0.25	*	−0.32	***	0.17	
**Covariates (CR)**						
Age	0.06	*	0.10	*	0.12	*
Gender	0.05	*	0.07	*	0.09	*
Homestay	−0.04	*	−0.09	*	−0.11	*

CG, caregiver; CR, care recipient/frail older adults; GR, both frail older adults and caregivers. *P < 0.05, **P < 0.01, ***P < 0.001.

In the first layer, shown in Table 3, a higher level of caregivers' QoL was predicted when there were a few more facilities providing social services (*B* = −0.03, *P* = 0.034), more grocery shops and markets (*B* = −0.01, *P* = 0.009), a shorter distance to the nearest metro station (*B* = 0.11, *P* = 0.012), and more greenery exposure (*B* = −0.69, *P* = 0.023). A higher level of QoL can be observed among caregivers who were older (*B* = −0.02, *P* < 0.001), or males (*B* = −0.66, *P* < 0.001). More serve use was associated with more facilities (*B* = 0.14, *P* = 0.019), more grocery (*B* = 0.12, *P* = 0.045), and a shorter distance to an adjacent metro station (*B* = −0.31, *P* = 0.037). Education attainment was insignificantly associated with QoL when controlling for other variables. Service use condition by both older adults and their caregivers were associated with stronger needs for services (*B* = 0.09, *P* < 0.001), higher caregiving stress (*B* = 0.01, *P* = 0.03). More frequent utilization of services was associated with older adults who were relatively older (*B* = 0.06, *P* = 0.012), female (*B* = −0.05, *P* = 0.033), and living independently (*B* = −0.03, *P* = 0.045).

In the second layer, shown in Table 4, outcome variables were found associated with intermediate and covariate variables. First, a lower frailty level of older adults was associated with a better QoL (*B* = 0.55, *P* < 0.001), and a higher frequency of service use (*B* = −0.25, *P* = 0.044), younger age (*B* = 0.06, *P* = 0.022), females (*B* = 0.05, *P* = 0.012), or living together with family members (*B* = −0.04, *P* = 0.006). Second, a better ADL functional level was associated with a better QoL (*B* = 0.25, *P* < 0.001), and a higher frequency of service use (*B* = −0.32, *P* = 0.028), younger age (*B* = 0.10, *P* = 0.028), females (*B* = 0.07, *P* = 0.042), or living together with family members (*B* = −0.09, *P* = 0.010). Last, a better IADL functional level was associated with a better QoL (*B* = 0.34, *P* < 0.001), and a lower frequency of service use (*B* = 0.17, *P* = 0.045), younger age (*B* = 0.12, *P* = 0.038), females (*B* = 0.09, *P* = 0.031), or living together with family members (*B* = −0.11, *P* = 0.049).

## Discussion

The underlying theoretical framework addressing our research questions was developed based on an integration of the Andersen's Model and the Stress-Adaptation Model including layers of critical components that predict the multi-dimensional health outcome of frail older adults. The Andersen's Model assumes that available services that meet the basic needs of frail older adults and their caregivers are beneficial to their frailty and activities of daily living (20). Application of the Stress-Adaptation Model in aging studies emphasizes the caregiver's role in the caregiving scenario living with a frail older adult and identifies the importance of caregiving stress on the health of older adults (42). Our findings fill in the theoretical gap. First, our findings add new knowledge and extend the application of the Andersen's Model by proposing that the neighborhood built environment factors serve an equivalent weight as individual factors in our proposed framework. Specifically, our findings indicate that the provision of greenery, the feasibility of transit, the density of grocery and social service facilities all evidently improve the acceptance rates of service provided, consequently affecting frail older adults' health outcomes (12). Second, our proposed framework explains the health model more powerfully by involving frail older adults' caregivers. The integration of the Stress-Adaptation Model avoids biased conclusion that can be potentially generated from the Andersen's Model concerning only the party of frail older adults regarding their health conditions. This proposed theoretical framework helps us easily appreciate the daily living scenarios, in which frail older adults and caregivers are generally interdependent. This framework best interprets why home-dwelling frail older adults rely heavily on family caregivers to facilitate their daily lives and promote desirable health status (45).

Policy implications for urban planning underscore the use of transit and greenery in neighborhoods and communities. To begin with, public transportations within walking distance should be the priority in urban planning in encouraging service use and enhancing QoL for community dwellers, who depend on transportations to facilitate service utilization and daily shopping for the maintenance of their basic needs. Both older adults and caregivers have substantially more frequent service use when convenient metro or bus stations are available near their homes (55). Improved QoL is found among older adults who have a desirable transit network, whereas scarcity of necessary transportations near home or close to destinations leads to impaired QoL and a lower potential to meet the needs for daily activities of frail older adults (56–58). In future urban planning for the aging communities with a high density of frail older adults, new bus stops/routes can be established and elevator-assisted metro exists built close to the frail population in order to facilitate their access to services that address their daily needs and health issues (59). Second, sustainable planning on urban greenery improves people's QoL. Humans are naturally attached to environmental greenery, which brings a sedative state and uplifts one's subjective mood. Empirical studies discover that greenery is vital in improving caregivers' life quality, enhancing their care quality, and further promoting frail older adults' health (13, 60). Green spaces also promote the quality of physical activities, such as cycling, hiking and tai chi, and positively reinforce more “green activities” (61, 62). Policymakers in future urban planning can involve environmental greenery and transit for sustainable service delivery and community development. Future environmental greenery planning should extend to small open spaces in the communities and even with the indoor settings in service facilities, which benefit both frail older adults and caregivers.

Our findings inform practices in social services. Improving service use is crucial in preventing frailty and difficulties in daily living. Neighborhood service facilities are imperative to address public health issues and to promote a sustainable age-friendly society. For instance, early screening and home-visiting services provided by the communities are the most fundamental priorities before any long-term services can be scheduled and delivered to the people in need. Accessible early screening for dwelling frail older adults can detect early signs of chronic diseases (e.g., cognitive impairment) that compromises one's capacity in ADL and IADL (e.g., dining, showering, walkability, etc.) and raises risks of emotional exhaustion, incontinence and accidental falls (49, 63). Household or home-visiting services help identify chronic diseases in a timely manner and confirm potential needs for community services. Neighborhood rehabilitation centers are also beneficial in meeting various urgent disease-related needs (17). Service use also benefits families of frail older adults and their caregivers altogether. Improved QoL indicating considerate self-care empowers caregivers to better care for their family members (including frail older adults). Neighborhood daycare and respite centers allow caregivers to have more leisure time beyond their daily care and improve the quality of life and care performance. Support groups sharing similar experiences among caregivers alleviate their emotional breakdown and empower their role identity in providing care. Sustainable social policies should promote diverse and useful social services to meet the basic needs of frail older adults in their daily living activities, followed by training sessions for community workers to ensure that services can be saturated in the target communities. Current social services should still emphasize meeting ADL needs rather than IADL needs as our findings indicate no association between service use and frail older adults' IADL. More advanced community services, such as commute services or financial education services, are not of maximum benefits when the frail older adults are having trouble walking outside freely, taking transportations, or engaging in services that require basic commuting and traveling abilities. Therefore, more basic services, such as home-visiting daycare, chores, and rehabilitation services are needed to support one's ADL difficulties in the preliminary stage of community services.

Among all the limitations in this study, a small sample size being the biggest challenge limits the capacity of making a comparison analysis between gender subgroups, or separately investigating the service use model in populations of frail older adults and caregivers, respectively. Moreover, the measurement of the neighborhood built environment does not address a full spectrum of characteristics of these environmental elements. For instance, distances to the service facilities and grocery stores can be recorded for analysis as this information determines the feasibility of the services provided in the communities. Furthermore, the greenery is found with its significance, yet it is surprisingly not associated with service use. This phenomenon could be due to the fact that the nature of the existing social services does not rely much on environmental greenery. Further investigation is necessary to explore how small green spaces, such as community cornered green or plants in facilities, can stimulate people in need to use services more frequently. Last, the generalizability of this study might be questionable if no future studies validate the theoretical framework in societies of other cultural or social backgrounds (64–68).

## Conclusion

Given that the neighborhood built environment factors are absent from the existing knowledge of frail older adults' health status, this study, conducted in Hong Kong with a sample size of 969, uses path analysis to investigate the significance of the urban planning for developing the neighborhood built environment and promotion in service uses. The analysis adopts a comprehensive perspective using the Andersen's health model for frail older adults and the Stress-Adaptation Model for caregivers. The results indicate the neighborhood built environment plays a critical role in promoting the frailty level and assisting capacities for daily living for frail older adults. In particular, more facilities offering services and groceries, a shorter distance to the nearest metro station, and more greenery exposure are associated with a better-expected health condition among frail older adults. In addition, service utilization and caregivers' QoL are indispensable to bridge the limited knowledge for us to understand how the neighborhood built environment can affect one's health. Implications are suggested to involve transit and greenery for urban planning policies, and involve more home-visiting services in the community practice.

## Data Availability Statement

The availability of the dataset presented analyzed in this study is not readily available because it belongs to the projects in Kerry Group Service Limited and Sau Po Centre on Ageing. Requests to access these datasets should be directed to Shuangzhou Chen, chenshuangzhou@hku.hk.

## Ethics Statement

The studies involving human participants were reviewed and approved by Human Research Ethics Committee for Non-Clinical Faculties from the University of Hong Kong, Hong Kong (E1808017). The patients/participants provided their written informed consent to participate in this study.

## Author Contributions

SC is responsible for conceptualization, formal analysis, investigation, methodology, project administration, preparing the original draft, and revisions. TW is responsible for conceptualization, investigation, reviewing, and editing. ZB is responsible for conceptualization, investigation, methodology, project administration, reviewing, and editing. VL is responsible for funding acquisition, data curation, and supervision. All authors agree to be accountable for the content of the work. All authors contributed to the article and approved the submitted version.

## Funding

This study received funding from the Hong Kong Kerry Group Service Limited (RS180122). The funder was not involved in the study design, collection, analysis, interpretation of data, and the writing of this article or the decision to submit it for publication. The funder had the following involvement with the study: evaluating the original commissioned grant research titled “The profile and service needs of elderly carers.” The funder had not involved in monitoring, evaluating, or providing further funding for this study.

## Conflict of Interest

VL, director of Center of Aging, in collaboration with the Hong Kong Council of Social Service were commissioned by the Hong Kong Kerry Group. The remaining authors declare that the research was conducted in the absence of any commercial or financial relationships that could be construed as a potential conflict of interest.

## Publisher's Note

All claims expressed in this article are solely those of the authors and do not necessarily represent those of their affiliated organizations, or those of the publisher, the editors and the reviewers. Any product that may be evaluated in this article, or claim that may be made by its manufacturer, is not guaranteed or endorsed by the publisher.
